# Loss of Dioxin Response Element-Mediated Induction of PKM2 Reprograms Hepatic Metabolism in Response to TCDD

**DOI:** 10.3390/ijms262210853

**Published:** 2025-11-08

**Authors:** Karina Orlowska, Rance Nault, Tim Zacharewski

**Affiliations:** 1 Department of Biochemistry & Molecular Biology, Michigan State University, East Lansing, MI 48824, USA; orlowska@msu.edu; 2 Institute for Integrative Toxicology, Michigan State University, East Lansing, MI 48824, USA; naultran@msu.edu; 3 Department of Pharmacology and Toxicology, Michigan State University, East Lansing, MI 48824, USA

**Keywords:** liver, TCDD, PKM2

## Abstract

2,3,7,8-Tetrachlorodibenzo-*p*-dioxin (TCDD) reprograms central carbon metabolism by switching pyruvate kinase expression from isoform M1 (*Pkm1*) to M2 (*Pkm2*), mediated by aryl hydrocarbon receptor (AhR) binding to a dioxin response element (DRE) located between exons 3 and 4 within the Pkm locus. To further investigate the consequences of *Pkm* isoform switching in TCDD elicited hepatotoxicity, we examined gene expression in primary hepatocytes isolated from mice with the Pkm locus DRE excised (Pkm^ΔDRE^). Wild-type and Pkm^ΔDRE^ hepatocytes were treated with 10 nM TCDD for 2, 4, 8, 12, 24, 48, 72, 96 and 120 h. Central carbon metabolite changes were also assessed in WT and Pkm^ΔDRE^ mice treated with 30 µg/kg TCDD every 4 day for 28 days. While AHR target genes were comparably induced, some genes exhibited divergent expression patterns in Pkm^ΔDRE^ mice compared to wild-types following treatment with TCDD. Notably, antioxidant gene expression was delayed in Pkm^ΔDRE^ hepatocytes. Metabolomic analysis also revealed differences in glycolytic, TCA cycle and pentose phosphate pathway metabolite levels in TCDD-treated WT and Pkm^ΔDRE^ liver extracts. In addition, amino acid metabolism and serine/glycine synthesis were also elevated, especially in Pkm^ΔDRE^. These findings indicate PKM2 induction affects the transcriptional and metabolic coordination of hepatic responses to TCDD.

## 1. Introduction

The biological consequences of dioxin-like compound (DLC) exposure are governed not only by extrinsic factors such as dose, exposure route, and duration, but also host factors such as genetic predisposition, epigenetic regulation, and systemic physiological context [1]. At the cellular level, defense mechanisms are rapidly engaged in response to DLCs to mitigate damage and restore homeostasis. This includes the induction of phase I and II enzymes, which facilitate biotransformation and elimination [2]. Additionally, immune cells are recruited to clear injured or dying cells, with metabolic pathways reprogramed to support antioxidant defenses, such as increased glutathione synthesis and recycling [3]. The coordinated orchestration of these processes results in diverse physiological outcomes ranging from adaptive repair to inflammation and other adverse consequences. Moreover, DLCs alter gene expression and enzymatic activity, disrupting endogenous metabolic networks [4]. A comprehensive elucidation of the underlying mechanisms of cellular defense and adaptation, may identify novel vulnerabilities and survival strategies that could be used to improve drug safety and environmental toxicology.

Among the DLCs, 2,3,7,8-tetrachlorodibenzo-*p*-dioxin (TCDD) is recognized as the most potent and well-studied congener, exerting its biological effects primarily through binding to the aryl hydrocarbon receptor (AhR), a basic helix-loop-helix ligand activated transcription factor [5]. TCDD passively diffuses into cells and binds to the cytosolic AhR, forming a ligand-receptor complex that translocates into the nucleus, where it heterodimerizes with the AhR nuclear translocator (ARNT). In the canonical pathway, the liganded complex then binds specific DNA motifs known as dioxin response elements (DREs) containing the core sequence 5′-GCGTG-3′ [6,7]. Other studies report liganded AHR-ARNT complex also binding to nonconsensus sites to elicit differential gene expression [6,7].

Beyond the well characterized role of inducing cytochrome P4501A1, TCDD is linked to the dose-dependent disruption of hepatic carbohydrate, lipid and amino acid metabolism. Notably, pyruvate kinase (PK), a key regulatory enzyme that catalyzes the conversion of phosphoenolpyruvate (PEP) to pyruvate, is significantly affected [8]. PK is encoded by two genes: *Pklr*, which gives rise to liver (PKL) and erythrocyte (PKR) isoforms, and *Pkm*, which produces two splice variants—PKM1 and PKM2—through mutually exclusive splicing of exons 9 and 10, respectively [9]. The PKM1 isoform is constitutively active and predominantly supports high rates of glycolysis, while PKM2 is subject to allosteric regulation and exists in a dynamic equilibrium between an active tetramer and a less active dimer form [10,11]. PKM2 has also been localized to the mitochondria and nucleus, where it contributes to transcriptional regulation and broader metabolic control [12,13]. These diverse functions of PKM2 underline its significance as a metabolic hub altered by TCDD exposure, with implications for both energy homeostasis and gene expression.

In addition to inducing PKM2 expression in mice, TCDD also increases the levels of upstream glycolytic intermediates, many of which can be rerouted to other metabolic pathways such as the pentose phosphate pathway (PPP) and serine/glycine biosynthesis. These pathways are important for maintaining redox homeostasis [14,15]. The diversion of glycolytic intermediates toward these pathways supports antioxidant defenses by promoting glutathione (GSH) synthesis and oxidized glutathione (GSSG) recycling in response to increased reactive oxygen species (ROS) levels induced by TCDD [16,17]. ChIP-seq, ChIP-chip, and ChIP-PCR assays previously identified AHR binding within an intronic region of the *Pkm* locus containing a canonical DRE core motif (5′-GCGTG-3′) [15]. Further, the functionality of this DRE was confirmed, as ChIP-PCR analysis demonstrated loss of AHR binding in hepatocytes from genetically engineered model, lacking the DRE sequence in the *Pkm* locus (Pkm^ΔDRE^ mice) [8]. The current study used Pkm^ΔDRE^ mice to further investigate the hepatic effects of TCDD on gene expression and metabolomic changes in the absence and presence of PKM2 induction.

## 2. Results

### 2.1. Pkm^ΔDRE^ Model Evaluation

**Figure 1:**
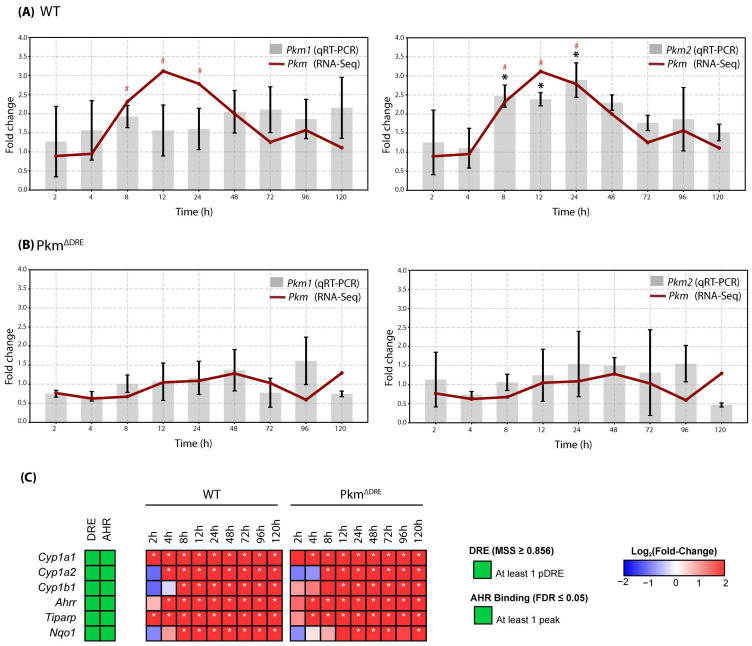
Pkm2 induction by TCDD in WT and Pkm^ΔDRE^ primary hepatocytes. *Pkm* expression was assessed by RNA-Seq (presented as red line graph) and qRT-PCR (presented as bar graph) in (**A**) WT and (**B**) Pkm^ΔDRE^ mouse hepatocytes treated with DMSO (vehicle) or 10 nM TCDD for 2, 4, 8, 12, 24, 48, 72, 96 and 120 h. Bars represent mean ± SD. Asterisk (qRT-PCR) or pound sign (RNA-Seq) indicates *p* ≤ 0.05 in fold change as determined by a two-way ANOVA and Tukey’s post hoc test. (**C**) Effect of TCDD on AhR battery genes. The presence of dioxin response elements (DRE) within the gene *loci* and in vivo AHR genomic enrichment at 2 h, following TCDD exposure (green). Color scale represents the log_2_ (fold change) for differential gene expression determined by RNA-Seq analysis (n = 5). The asterisk indicates differential expression with a posterior probability (P1 (t)) ≥ 0.80.

To assess the role of Pkm2 expression in response to TCDD, a time-course study was performed using primary hepatocytes isolated from WT controls and Pkm^ΔDRE^ mice lacking a functional DRE within the Pkm loci [8]. Isolated hepatocytes were treated with 10 nM TCDD or DMSO vehicle and were harvested at multiple time points. RNA-Seq analysis revealed a time- and genotype-dependent gene expression response to TCDD between WT and Pkm^ΔDRE^ hepatocytes. In WT hepatocytes, qRT-PCR confirmed TCDD induced *Pkm2*, beginning at 8 h that reached a maximum at 24 hrs and persisted through 48 h, with no significant changes in *Pkm1* expression at any time point. Although RNA-Seq data suggested an overall increase in *Pkm* transcript levels between 8 and 24 h, this reflects the induction of *Pkm* as opposed to a specific isoform (Figure 1A,B). The induction of known AHR responsive genes (*Cyp1a1*, *Cyp1a2*, *Cyp1b1*, *Ahrr*, *Tiparp*, *Nqo1*) were comparable in hepatocytes isolated from Pkm^ΔDRE^ and WT mice (Figure 1C). These genes contain functional DREs within their regulatory regions. AHR genomic binding at these DREs has been previously confirmed, underscoring their AHR-dependent regulation.

### 2.2. Effects of TCDD on Gene Expression in Cultured Mouse Primary Hepatocytes (In Vitro)

**Figure 2:**
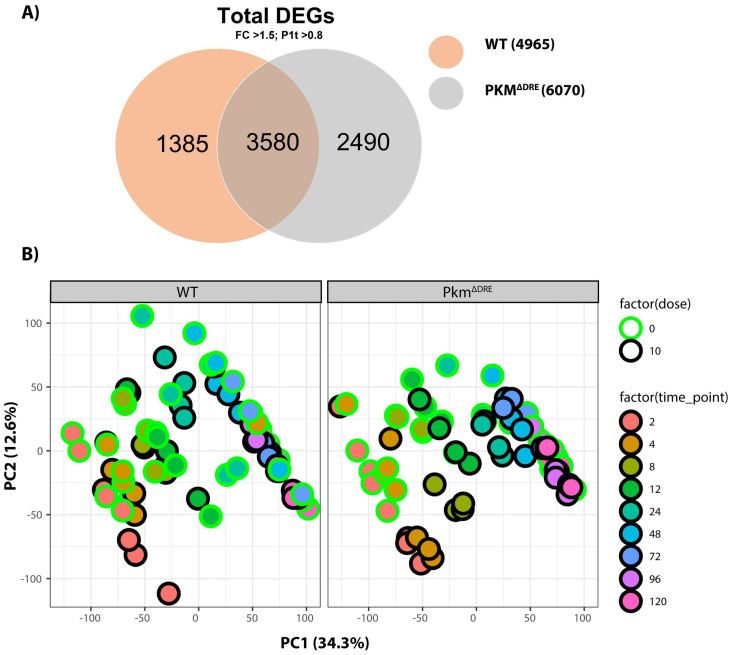
Differentially expressed genes (DEGS) in primary mouse hepatocytes treated with 10 nM TCDD across all time points. (**A**) DEGS in primary mouse hepatocytes treated with 10 nM TCDD across all time points. (**B**) Principal component analysis (PCA). Time-dependent gene expression was assessed in WT and Pkm^ΔDRE^ primary mouse hepatocytes treated with DMSO vehicle or 10 nM TCDD. Differential gene expression (P1 (t)) ≥ 0.80 and fold change (FC) ≥ 1.5) was determined at 2, 4, 8, 12, 24, 48, 72, 96, and 120 h.

We next examined the effects of TCDD on hepatocyte gene expression using RNA-Seq. Principal component analysis (PCA) demonstrated progressive temporal separation of TCDD-treated WT and Pkm^ΔDRE^ hepatocytes (Figure 2B). In total, 4965 and 6070 differentially expressed genes (DEGs, P1 (*t*) > 0.80 and fold change > 1.5) were detected in WT and Pkm^ΔDRE^ hepatocytes, respectively. Approximately 3580 DEGs were in common with 1385 and 2490 unique to WT and Pkm^ΔDRE^ hepatocytes, respectively (Figure 2A). However, the timing and magnitude of differential expression varied markedly between WT and Pkm^ΔDRE^ hepatocytes. WT hepatocytes exhibited the greatest number of DEGs at 2 h with the majority being induced (Table 1). In contrast, maximum differential gene expression in Pkm^ΔDRE^ hepatocytes was delayed to 8 h (Table 1). After 12 h, the number of DEGs was comparable in both WT and Pkm^ΔDRE^ hepatocytes.

Observed differences between DEG numbers within timepoints could not be simply explained by a temporal shift in gene expression. At 2 h, TCDD elicited 1848 DEGs in WT hepatocytes compared to 756 in Pkm^ΔDRE^ hepatocytes, whereas at 8 h only 303 were differentially expressed in WT compared to the 2772 DEGs in Pkm^ΔDRE^ hepatocytes, with only 348 genes overlapping between the two groups (Table 1, Supplementary Materials Figure S2). Therefore, transcriptional responses are not merely delayed or shifted in time but reflect different expression dynamics with different patterns of dysregulation. For example, *Ldha* and *Ldhb* had opposing expression patterns, while canonical AHR target gene induction was comparable in WT and Pkm^ΔDRE^ hepatocytes (Figure 1C). Notably, antioxidant genes such as *Gsta2*, *Gsta4*, *Gstm2*, *Cbr3*, and *Pmm1* were delayed or had attenuated induction in Pkm^ΔDRE^ hepatocytes (Supplementary Materials Figure S1B), suggesting an extended PKM2 role in antioxidant defense beyond metabolic reprograming to provide intermediates for glutathione biosynthesis and NADPH to recycle oxidized glutathione [8,15]. Functional analysis revealed that genes upregulated at 2 h in WT were enriched for RNA biosynthetic and metabolic processes, as well as DNA-templated transcription, further implicating a nuclear role for PKM2 in regulating gene expression (Supplementary Materials Figure S1A) [18]. In contrast, Pkm^ΔDRE^-specific gene expression at 8 h was enriched for cellular organization and cytoskeletal remodeling, reflecting the induction of a fundamentally different transcriptional program (Supplementary Materials Figure S1A), suggesting the absence of the nuclear PKM2 function disrupts early transcriptional coordination in response to TCDD. Together, these results indicate that PKM2 is an important modulator of AHR-mediated gene expression, influencing not only the timing but also hepatocyte gene expression programs.

**Figure 3:**
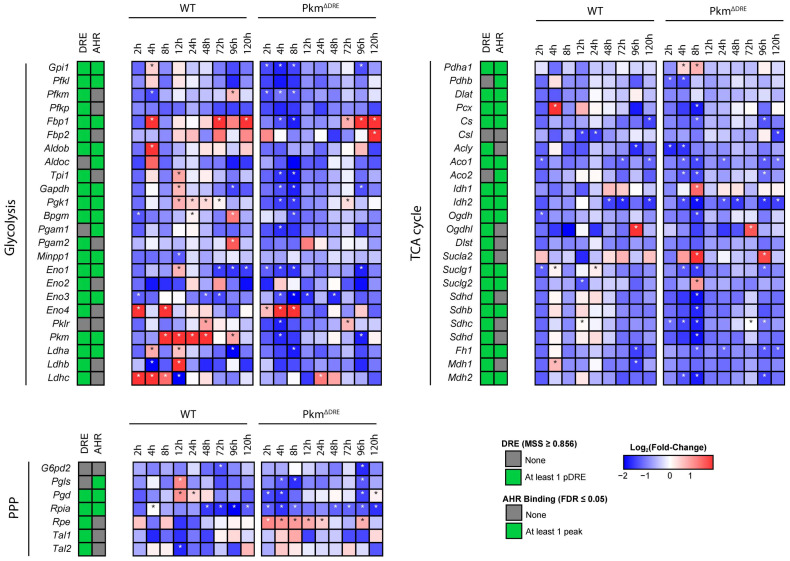
Effect of TCDD on glycolysis, PPP and TCA cycle gene expression. The presence of putative dioxin response elements (pDRE) within the gene loci and in vivo hepatic AHR genomic enrichment at 2 h, following TCDD treatment (green). Time-dependent gene expression was assessed in wild type (WT) and Pkm^ΔDRE^ primary mouse hepatocytes treated with DMSO (vehicle) or 10 nM TCDD for 2, 4, 8, 12, 24, 48, 72, 96 and 120 h. Color scale represents the log_2_ (fold change) for differential gene expression determined by RNA-Seq analysis (n = 5). The asterisk indicates differential expression with a posterior probability (P1 (t)) ≥ 0.80.

We further analyzed differential gene expression specifically in glycolysis, pentose phosphate, and tricarboxylic acid (TCA) pathways. Among glycolytic genes *Pfkl*, *Pfkp*, *Aldob*, *Pgam1* and *2* were either modestly induced or remained unchanged in both genotypes. Other glycolytic genes (*Gpi1*, *Aldoa*, *Tpi1*, *Gapdh*, *Pgk1*, *Ldha* and *Ldhb*) were induced in WT hepatocytes at early time points while exhibited no induction or were repressed in Pkm^ΔDRE^ following treatment with TCDD (Figure 3). *Pgls* and *Pgd,* genes associated with the oxidative phase and NADPH production in the PPP, were induced at 12 h in WT hepatocytes, but repressed in Pkm^ΔDRE^ hepatocytes (Figure 3). *Rpia*, which participates in the non-oxidative arm of the PPP, was consistently downregulated in both WT and Pkm^ΔDRE^ hepatocytes while *Rpe* was induced in Pkm^ΔDRE^ hepatocytes with no change in WT. This genotype-specific expression may reflect compensatory responses in the absence of sufficient PPP activity. TCDD also repressed TCA cycle gene expression in Pkm^ΔDRE^ hepatocytes. At 8 h, *Aco1*, *Aco2*, *Idh2*, *Sdhb*, *Sdhc*, *Sdhd* and *Mdh2* were either unchanged or repressed compared to WT hepatocytes while *Idh1*, *Sucla2*, and *Suclg2* exhibited induction (Figure 3). Collectively, the differential effects of TCDD in WT and Pkm^ΔDRE^ hepatocytes further support a gene expression regulatory role for PKM2 in addition to its metabolic activities.

### 2.3. Effects of TCDD on Hepatic Metabolite Levels (In Vivo)

Our previous studies demonstrated that TCDD elicited different fibrosis responses in the liver of WT and Pkm^ΔDRE^ mice [8]. We therefore hypothesized that TCDD elicited gene expression changes in Pkm^ΔDRE^ hepatocytes would be reflected in altered hepatic metabolite levels. To test this, WT and Pkm^ΔDRE^ mice were orally gavaged with 30 ug/kg TCDD every four days for 28 days, a treatment regimen previously used to elicit hepatic fibrosis [8]. PCA showed the first two principal components captured 58.7% of the total variance, with PC1 (*x*-axis) accounting for 42.8% and PC2 (*y*-axis) explaining 15.9% of the variability (Supplementary Materials Figure S3). Separation along PC1 reflected TCDD treatment separating from sesame oil (control) irrespective of genotype. WT and Pkm^ΔDRE^ hepatocytes formed discrete clusters that separated along PC2 based on genotype (Supplementary Materials Figure S3) suggesting TCDD treatment is the primary driver of metabolic variance, while genotype further stratified response differences. More specifically, there were differences in redox- and amino acid-related metabolites, including oxidized glutathione, aspartate, glycine, serine, and glutamate, suggesting treatment-induced changes in amino acid metabolism and responses to increasing oxidative stress (Supplementary Materials Table S1). Malate, fumarate, acetyl-CoA, and ketoglutarate drove PC2 separation, implicating the TCA cycle and energy metabolism as key pathways involving regulation by PKM2.

**Figure 4:**
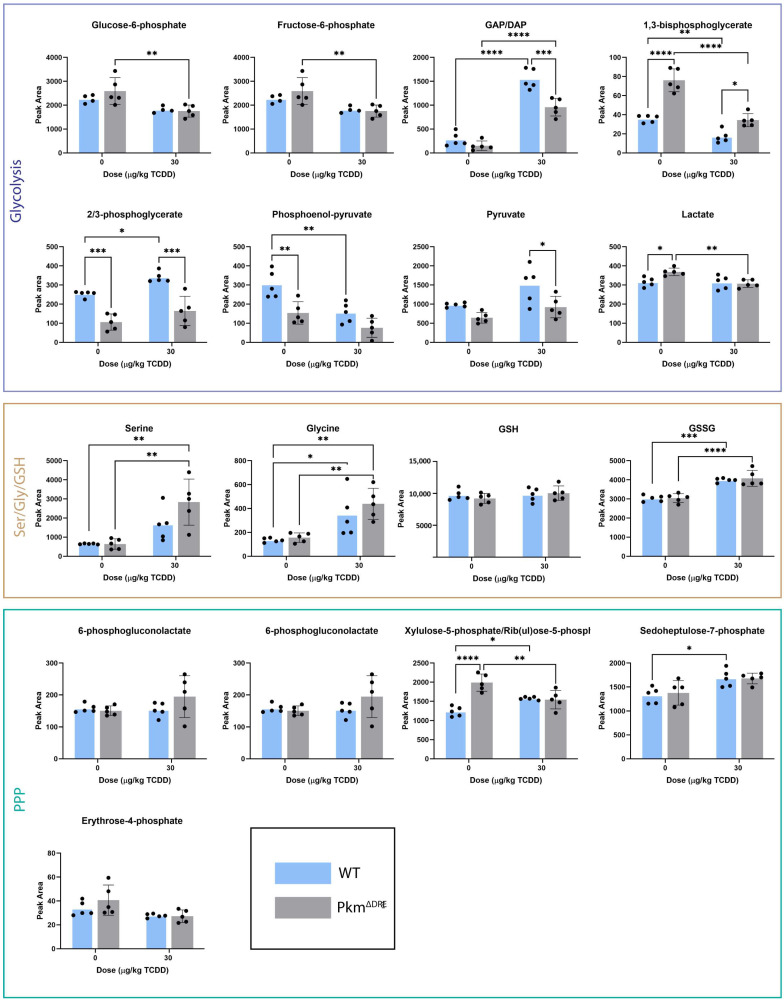
Levels of glycolytic, Ser/Gly/GSH and PPP metabolites. WT and Pkm^ΔDRE^ mice were treated with sesame oil (vehicle) or 30 µg/kg TCDD every 4 day for 28 days. Livers were snap-frozen, and metabolites were extracted using 80% methanol. Samples were analyzed on a Xevo G2-XS Quadrupole Time of Flight mass spectrometer attached to a Waters Acquity UPLC (Waters) operated in negative-mode electrospray ionization. Peak areas were quantified using MassLynx software V4.2. Bars represent mean ± SD. Asterisk indicates significance (* *p* ≤ 0.05, ** *p* < 0.01, *** *p* ≤ 0.001, **** *p* ≤ 0.0001) determined by two-way ANOVA and Tukey’s post hoc test (n = 5).

We also compared the levels of 28 central carbon metabolites between WT and Pkm^ΔDRE^ mice following oral gavage with TCDD. Of the eight glycolytic intermediates examined, four (glyceraldehyde-3-phosphate/dihydroxyacetone phosphate (GAP/DAP), 1,3-bisphosphoglycerate (1,3-BPG), 2/3-phosphoglycerate and phosphoenolpyruvate (PEP)) were altered by TCDD in WT liver extracts while five (glucose-6-phosphate (G-6-P), fructose-6-phosphate (F-6-P), GAP/DAP, 1,3-BPG, and lactate) were changed in Pkm^ΔDRE^ livers by TCDD (Figure 4). In WT liver extracts, levels of 1,3-BPG, 2/3-phosphoglycerate, and PEP were reduced while GAP/DAP levels were significantly elevated, indicating TCDD disrupted glycolytic flux. In Pkm^ΔDRE^ mice, G-6-P, F-6-P, 1,3-BPG and lactate were significantly decreased by TCDD, while GAP/DAP was markedly increased.

The levels of three out of five measured PPP metabolites remained unchanged following TCDD treatment (Figure 4). Increases in xylulose-5-phosphate (X-5-P) and sedoheptulose-7-phosphate levels were seen in WT liver extracts, whereas only X-5-P was decreased in Pkm^ΔDRE^ liver extracts.

The serine/glycine/glutathione biosynthesis pathway uses the glycolytic intermediate, 3-phosphoglycerate, as a substrate. Three of four profiled metabolites were altered: glycine and GSSG were elevated in WT mice, while serine, glycine, and GSSG were increased in Pkm^ΔDRE^ mice (Figure 4). Serine and glycine, GSH precursors, were higher in Pkm^ΔDRE^ when compared to WT, suggesting disrupted GSH biosynthesis in the absence of PKM2.

**Figure 5:**
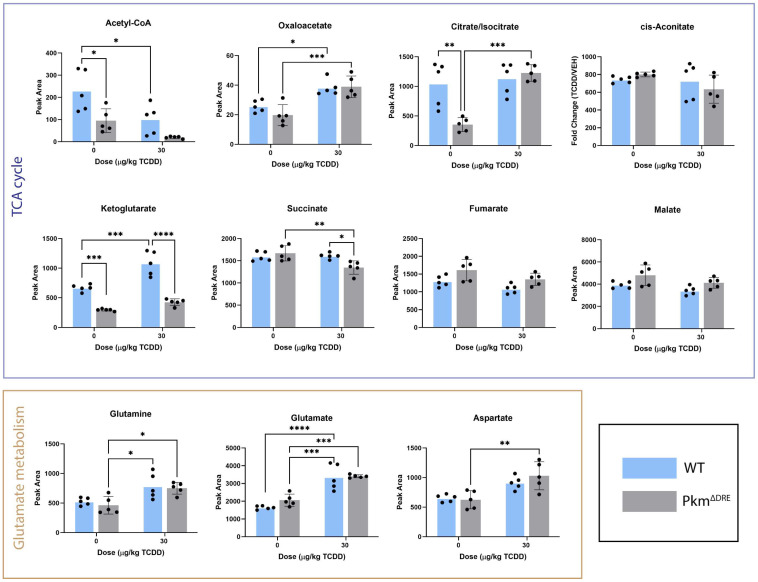
Levels of TCA cycle metabolites. WT and Pkm^ΔDRE^ mice were treated with sesame oil (vehicle) or 30 µg/kg TCDD every 4 day for 28 days. Livers were snap-frozen, and metabolites were extracted using 80% methanol. Samples were analyzed on a Xevo G2-XS Quadrupole Time of Flight mass spectrometer attached to a Waters Acquity UPLC (Waters) operated in negative-mode electrospray ionization. Peak areas were quantified using MassLynx software. Bars represent mean ± SD. Asterisk indicates significance (* *p* ≤ 0.05, ** *p* < 0.01, *** *p* ≤ 0.001, **** *p* ≤ 0.0001) determined by two-way ANOVA and Tukey’s post hoc test (n = 5).

Analysis of eight TCA cycle intermediates also revealed differences between genotypes (Figure 5). In WT livers, acetyl-CoA levels were decreased, while oxaloacetate and α-ketoglutarate were elevated, as previously reported [15]. Conversely, in Pkm^ΔDRE^ livers, succinate levels were decreased, and similar to WT, both oxaloacetate and α-ketoglutarate levels increased.

Finally, glutamine, glutamate and aspartate levels were all increased in Pkm^ΔDRE^ liver extracts, whereas only glutamate was elevated in WT (Figure 5). These changes in Pkm^ΔDRE^ liver extracts provides further support that the loss of PKM2 expression not only affects gene expression but also reprograms central carbon metabolism in response to TCDD treatment.

## 3. Discussion

In this study, we have demonstrated that PKM2 plays a critical role in modulating hepatocellular responses to TCDD at both the gene expression and metabolic levels. While TCDD induced *Pkm2* expression in WT hepatocytes, beginning at 8 h, and persisting until 48 h, we found induction absent in Pkm^ΔDRE^ hepatocytes. Despite comparable induction of AHR target genes (e.g., Cyp1a1, *Cyp1a2*, *Cyp1b1*) in both models, RNA-Seq analysis revealed marked divergence in the timing and magnitude of TCDD-induced gene expression. WT hepatocytes exhibited an early transcriptional response peaking at 2 h (1848 DEGs), whereas the maximal response in Pkm^ΔDRE^ hepatocytes occurred at 8 h (2772 DEGs), with minimal overlap in differentially expressed genes at corresponding time points. Notably, genes involved in antioxidant defense (*Gsta2*, *Gsta4*, *Gstm2*) were delayed or attenuated in Pkm^ΔDRE^ hepatocytes, suggesting PKM2 contributes to redox regulation in addition to supporting metabolic functions. Furthermore, loss of PKM2 altered the expression of key genes in glycolysis (*Gapdh*, *Pgk1*, *Ldha*, *Ldhb*), PPP (e.g., Pgls, *Pgd*), and the TCA cycle (*Aco1*, *Sdhb*, *Mdh2*), many of which were either repressed or failed to respond to TCDD in Pkm^ΔDRE^ hepatocytes. Similar changes were seen in in vivo metabolic shifts: Pkm^ΔDRE^ livers exhibited decreased levels of glycolytic intermediates (e.g., G-6-P, F-6-P, 1,3-BPG), altered PPP metabolites (reduced X-5-P), and dysregulation of TCA cycle components (e.g., decreased succinate and acetyl-CoA). Moreover, increased levels of serine, glycine, and GSSG in Pkm^ΔDRE^ mice pointed to disruption of homeostasis maintained by GSH.

AHR has emerged as a pivotal regulator of hepatic metabolic reprogramming, with roles in the development of metabolic dysfunction-associated fatty liver disease (MAFLD), that increases the risk for progression to end-stage liver disease, and hepatocellular carcinoma (HCC) [19,20]. A growing body of evidence implicates PKM2 as a downstream effector in AHR-mediated responses, including toxicological outcomes [15,21,22]. Consistent with this, our prior work demonstrated that TCDD as well as other AHR agonists including polychlorinated biphenyl (PCB)126, 2,3,7,8-tetrachlorofuran (TCDF), and β-naphthoflavone significantly induced PKM2 expression at both the mRNA and protein levels. In contrast, PCB153, a non-coplanar congener with no AHR binding affinity, did not elicit PKM2 induction, confirming AHR regulation of *Pkm2* expression [15,22]. This induction was independent of NRF2, as TCDD treatment still induced PKM2 in hepatocytes lacking NRF2 [23]. Chromatin immunoprecipitation (ChIP) studies further identified AHR genomic binding at a putative DRE (pDRE) located between exons 3 and 4 of the *Pkm* locus, suggesting direct transcriptional regulation. In Pkm^ΔDRE^ mutant mice, AHR binding was absent *Pkm* locus, but remained detectable at characterized DREs in known target genes such as *Cyp1a1*, *Lekr1*, and *Tiparp* [8]. Importantly, deletion of the 5′-GCGTG-3′ motif within this intragenic region abolished the TCDD-induced PKM2 upregulation in vivo. Moreover, TCDD did not induce PKM2 induction in primary hepatocytes isolated from AhR knockout mice, underscoring the necessity of the AHR [8]. Collectively, these results provide compelling evidence that AHR directly regulates PKM2 expression through a functional DRE element between exons 3 and 4, thereby contributing to the metabolic reprogramming elicited by TCDD.

To elucidate how AHR activation alters hepatic glucose metabolism, we examined glycolysis at both the gene expression and metabolite levels in WT and Pkm^ΔDRE^ models. In WT hepatocytes, early glycolytic genes such as *Gpi1*, *Aldoa*, *Tpi1*, *Gapdh*, *Pgk1*, *Ldha*, and *Ldhb* were transiently induced, while this response was absent or repressed in *Pkm*^ΔDRE^ cells, suggesting AHR was required for PKM2 induction. In WT liver extracts, 1,3-BPG, 2/3-phosphoglycerate, and PEP levels were reduced while GAP/DAP was significantly elevated by TCDD, indicating modulation of glycolytic flux. In Pkm^ΔDRE^ mice, G-6-P, F-6-P, 1,3-BPG and lactate were significantly decreased, while GAP/DAP was markedly increased. These changes extend previously reported effects of TCDD on glycolysis. For example, TCDD induced glucose transporter and glycolytic gene expression, leading to increased glucose uptake and lactate production in human primary hepatocytes [24]. Prior studies in female mice have also shown that PKM2 induction by TCDD resulted in the accumulation of glycolytic intermediates such as G-6-P and 3-PG. [15]. This metabolic bottleneck created by PKM2 induction decreases glycolytic throughput forcing accumulating upstream metabolites into ancillary pathways like PPP and serine/folate biosynthesis, both critical for NADPH generation as well as GSH biosynthesis and oxidized GSH recycling [15]. TCDD increased PPP flux as was evident by elevated levels of gluconolactone-P, ribulose 5-phosphate, and ribose 5-phosphate, along with reduced erythrose-4-phosphate and 5-phosphoribose 1-diphosphate. Furthermore, Heo et al. [25] also demonstrated that AHR activation supports mitochondrial homeostasis by enhancing PPP activity, an effect that was abrogated in AHR-knockout hepatocytes. Importantly, impaired antioxidant defenses has previously been reported in our Pkm^ΔDRE^ model [8] which is further supported by our findings. Although Pkm^ΔDRE^ livers exhibited reductions in G-6-P and F-6-P, this did not correspond with increased PPP metabolite levels. In contrast, WT livers showed elevated levels of xylulose/ribulose-5-phosphate and sedoheptulose-7-phosphate following TCDD treatment. These same metabolites were either unchanged or reduced in Pkm^ΔDRE^ mice, further highlighting the role of PKM2 in coordinating AHR-mediated regulation of glycolysis and PPP activity.

In WT livers, levels of 2/3-PG were elevated following TCDD treatment, whereas this response was absent in the Pkm^ΔDRE^ model. An increase in 3-PG has previously been observed in female mice exposed to TCDD [15]. 3-PG can be diverted to the serine biosynthesis pathway, where serine is subsequently converted into glycine, contributing to the generation of 5,10-methylene-tetrahydrofolate and the production of NADPH, a reducing equivalent used in the recycling of oxidized GSH. Serine/folate metabolism is estimated to contribute 10–40% of cellular NADPH production, comparable to the ~30% contribution from the PPP [26]. 3-PG did not accumulate in Pkm^ΔDRE^ mice, possibly due to its use for serine biosynthesis consistent with the increase in serine levels in the Pkm^ΔDRE^ model. Glycine was induced in both WT and Pkm^ΔDRE^ livers, though the increase was more prominent in Pkm^ΔDRE^ mice. While we did not report changes in GSH levels in the present study, due to the LC-MS method used here, a companion study used a more sensitive derivitization approach to more accurately measure GSH and GSSG levels to show that TCDD dose-dependently increased GSH in WT mice at both 10 μg/kg and 30 μg/kg doses [8]. In contrast, GSH only increased at 10 μg/kg dose in Pkm^ΔDRE^ mice [8]. Furthermore, GSSG increased in both genotypes at 30 μg/kg TCDD, but levels were two-fold higher in Pkm^ΔDRE^ mice, suggesting impaired recycling of GSSG to GSH in the absence of PKM2. These results are consistent with lower NADPH availability, impaired GSSG recycling, and disrupted GSH biosynthesis in the absence of PKM2 induction. In support of the proposed link between PKM2 induction and antioxidant defense, our previous study using WT and Pkm^ΔDRE^ primary hepatocytes demonstrated that loss of PKM2 increased susceptibility to oxidative stress. Pkm^ΔDRE^ hepatocytes co-treated with TCDD and hydrogen peroxide exhibited greater cytotoxicity (LC_50_ ≈ 290 μM) than WT cells (LC_50_ ≈ 620 μM), accompanied by lower induction of key antioxidant genes (*Gpx2*, *Gpx4*, *Sod1–3*) [8]. Consistent with these gene expression changes, CAT and SOD enzymatic activities were also lower in Pkm^ΔDRE^ livers relative to WT [8]. Together, these results provide direct biochemical support that AHR-dependent PKM2 induction is important in maintaining redox balance and antioxidant capacity following treatment with TCDD.

TCDD also reduced hepatic acetyl-CoA levels in WT mice, as previously reported, demonstrating that reduced glycolytic flux and inhibition of β-oxidation impacts energy metabolism [15,27]. Additionally, TCDD suppresses pyruvate dehydrogenase activity and downregulates the expression of *Acss2* and *Acly*, key enzymes involved in generating the cytosolic acetyl-CoA pool [27]. The observed increase in α-ketoglutarate levels in the current study, alongside elevated glutamate levels, suggests enhanced glutaminolysis as a compensatory mechanism to replenish TCA cycle intermediates and maintain ATP production despite the acetyl-CoA deficit. Intriguingly, this was not observed in Pkm^ΔDRE^ mice. Following TCDD treatment, acetyl-CoA levels remained unchanged, and instead, TCA flux appeared enhanced, as indicated by increased citrate and isocitrate levels. Despite increased glutamine and glutamate, α-ketoglutarate levels were stable, suggesting its downstream use. Accordingly, there were reductions in succinate while fumarate and malate were unchanged, indicating a change in TCA flux dynamics. Moreover, Pkm^ΔDRE^ livers showed higher levels of oxaloacetate and aspartate, suggesting increased input into the TCA cycle and higher aspartate production, possibly due to increased transamination. This implies that in the absence of PKM2 induction, hepatocytes may adopt an alternative metabolic strategy to maintain TCA activity and redox balance. Previous reports have shown that PKM2 loss or inactivation can promote oxidative metabolism through increased mitochondrial flux with increased glutamine utilization [11,28]. These findings are similar to the metabolic changes seen in Pkm^ΔDRE^ mice, suggesting that absent PKM2, mitochondrial metabolism may use alternative pathways like glutaminolysis to address reduced acetyl-CoA availability. However, these compensatory metabolic changes may not solely reflect the direct consequence of PKM2 loss but could also arise from broader AhR-mediated metabolic reprogramming. AhR activation regulates numerous pathways that influence mitochondrial function, coactivator recruitment, and secondary transcriptional regulators which can affect cellular energy and redox states. Therefore, interactions between PKM2 and indirect regulatory mechanisms within the AhR signaling network cannot be excluded. Further studies are warranted to determine whether compensatory metabolism is a direct result of PKM2 loss or secondary to broader metabolic reprogramming triggered by TCDD.

The comparison of short-term in vitro exposures in primary hepatocytes and long-term in vivo treatments in mice was intended to provide complementary insights into AHR-dependent regulation of *Pkm2*. Short-term studies in hepatocytes allowed for the characterization of direct gene expression responses following AHR activation, minimizing confounding influences from systemic metabolism or inter-cell/-tissue communication. In contrast, the mouse studies captured cumulative and adaptive effects of sustained AHR activation within a physiological context, reflecting the integration of hepatic and extrahepatic signaling pathways over time. However, several limitations should be acknowledged when comparing results between these systems. Differences in exposure duration, metabolic state, and cellular microenvironment may affect the amplitude and kinetics of AHR-dependent responses. In addition, the Pkm^ΔDRE^ model represents a whole-body deletion of the DRE element within *Pkm* loci, therefore indirect effects from extrahepatic tissues cannot be ruled out. Nonetheless, the induction of *Pkm2* and associated metabolic reprogramming observed specifically in hepatocytes indicate that the effects reported here largely reflect intrinsic, AHR-dependent mechanisms.

Collectively, these results establish PKM2 as a regulator of AHR-mediated transcriptional and metabolic reprogramming in hepatocytes exposed to TCDD. Loss of PKM2 induction led to temporal and functional differences in gene expression, particularly in pathways associated with antioxidant defense, glycolysis, PPP, and the TCA cycle. Metabolomic analyses confirmed that these transcriptional changes translate into altered metabolite levels, with Pkm^ΔDRE^ livers displaying impaired glycolytic flux, disrupted NADPH-generation, and TCA cycle activity suggestive of increased glutaminolysis and transamination to sustain energy and redox homeostasis. These findings underscore dual roles for PKM2 in coordinating gene expression and metabolic adaptation in response to TCDD and supports a model in which PKM2 functions not only as a metabolic enzyme but also as a transcriptional co-regulator.

## 4. Materials and Methods

### 4.1. Pkm^ΔDRE^ Model Generation

Pkm^ΔDRE^ mice lacking a functional DRE within the Pkm loci were generated as described previously [8]. In brief, the intronic DRE site (mm10: chr9: 59,668,103–59,668,107) was removed using CRISPR–Cas9 genome editing to generate a Pkm^ΔDRE^ mutant mouse line. To achieve this, a guide RNA (gRNA; 5′-AGGAAGTGGTTATGAAAGCAGGG-3′) targeting the intronic DRE sequence upstream of exon 4 was designed to introduce a double-stranded break 9 bp upstream of the motif. A single-stranded oligodeoxynucleotide (ssODN) repair template was used to delete a 5′-GCGTG-3′ motif and introduce a G→A substitution in the PAM sequence, preventing further Cas9 activity. The DRE deletion was confirmed by Sanger sequencing, and founders were backcrossed with C57BL/6N WT mice for eight generations. Homozygous Pkm^ΔDRE^ mice were subsequently established and displayed no abnormal phenotypic or behavioral traits. These mutant mice were indistinguishable from WT controls in terms of growth, diet, and reproductive performance. WT controls were generated through backcrossing with C57BL/6N mice.

### 4.2. Primary Hepatocytes Isolation and Culture

Primary hepatocytes were isolated from WT and Pkm^ΔDRE^ mice using a standard perfusion and collagenase digestion protocol [29]. Briefly, the liver was first perfused via the inferior vena cava with calcium- and magnesium-free Hanks’ Balanced Salt Solution (HBSS; Sigma-Aldrich, St. Louis, MO, USA) containing 0.5 mM EGTA, 5.5 mM glucose, and penicillin-streptomycin followed HBSS supplemented with 1.5 mM calcium chloride, 5.5 mM glucose, antibiotics, and 0.02% (*w*/*v*) type IV collagenase (Sigma-Aldrich). The liver was then excised, gently dissociated in 10 mL of William’s Medium E (WME; Invitrogen, Carlsbad, CA, USA), and filtered to remove tissue debris and aggregates. Hepatocytes were collected by centrifugation and washed three times with WME. Only cell preparations with ≥90% viability, assessed via trypan blue exclusion, were used.

Hepatocytes were seeded at a density of 1 × 10^6^ cells/well on type I collagen-coated 6-well plates and cultured in WME supplemented with 5% fetal bovine serum (FBS), 1% penicillin/streptomycin, 1% Glutamax, 0.3 mM ascorbic acid, and 100 nM dexamethasone. After a 3 h attachment period, non-adherent cells were removed and media was replaced with fresh WME containing 1% ITS Premix, antibiotics, Glutamax, ascorbic acid, and dexamethasone with DMSO vehicle or 10 nM TCDD and maintained at 37 °C in a 5% CO_2_ incubator.

### 4.3. RNA Isolation and Quantitative Real-Time Polymerase Chain Reaction (qRT-PCR)

Primary mouse hepatocytes (1 × 10^6^ cells) were plated in 6-well plate for RNA isolation. Medium was removed and cells were rinsed two times with cold PBS. While on ice, 1 mL of TRIzoL reagent was added to each well and plates were scraped. RNA was extracted using an additional 5:1 phenol–chloroform step (Sigma Aldrich, St. Louis, MO, USA). RNA quantity and purity (260/280 ratio) was assessed with a NanoDrop 1000. Total RNA was converted to cDNA with SuperScript II (Invitrogen, Carlsbad, CA, USA) using oligo dT primer. qRT-PCR was performed using iQ SYBR Green Supermix (BioRad, Hercules, CA, USA) on a Bio-Rad CFX Connect Real-Time PCR Detection System. Gene expression relative to vehicle control was calculated using the 2^−ΔΔCT^ method, where each sample was normalized to the geometric mean of *Actb*, *Gapdh*, and *Hprt*. SYBR green Mastermix (Life Technologies, Carlsbad, CA, USA) was used to analyze relative gene expression. The primer sequences are listed in Supplementary Table S2.

### 4.4. RNA-Seq

Sequencing were performed by Novogene on NovaSeq 6000 for a total of 20 M 150 base-pair unstranded paired-end reads per sample. Alignment to GRCm39 (release 104) was performed with STAR (v2.7.3a) [30] after quality control using FastQC (v0.11.7) trimming adaptors with Trimmomatic (v0.39) [31]. Gene counts underwent variance stabilization transformation using DESeq2 (v1.42.1) (https://doi.org/10.1186/s13059-014-0550-8) in R (v4.3.2). Posterior probabilities (P1 (t)’s) were calculated using an empirical Bayes method [32] accounting for time-dependent changes compared to time-matched controls. Genes with a |fold-change| ≥ 1.5 and P1 (t) ≥ 0.8 were considered differentially expressed. Principal components analysis was performed in R using prcomp from the stats base package. Functional over-representation analysis was performed using an updated collection of gene sets form the Gene Set Knowledgebase (GSKB) and the bc3net for sets containing between 10 and 500 genes. A more stringent filtering criteria was used for functional enrichment analysis (|fold-change| ≥ 2.0 and P1 (t) ≥ 0.9) to identify more perturbed pathways. Figures were generating using ggplot2 (v3.4.4) and formatted for style using Adobe Illustrator Version 16.0.0. Data are deposited in the Gene Expression Omnibus (accession ID GSE309637).

### 4.5. Treatment Regimen

Male WT and Pkm^ΔDRE^ mice were housed in Innovive Innocages (San Diego, CA, USA) lined with ALPHA-dri bedding (Shepherd Specialty Papers, Chicago, IL, USA), maintained under controlled conditions (23 °C, 30–40% humidity, 12-h light/dark cycle), with unrestricted access to Harlan Teklad Rodent Diet 8940 (Madison, WI, USA) and water. Eight animals were assigned to each treatment group. Beginning on postnatal day (PND) 30–32, at weight 13–16 g, mice were orally gavaged with sesame oil (vehicle, 100 µL) or 30 μg/kg TCDD (in sesame oil, 100 µL) every four days for 28 days (total of 7 doses), between zeitgeber time (ZT) 0–3. Animals were euthanized by overdose of carbon dioxide followed by cardiac puncture to ensure death before tissue collection, in accordance with approved institutional animal care and use protocols. Following euthanization, livers were excised, weighed, snap frozen in liquid nitrogen, and stored at −80 °C. The TCDD dose levels and treatment regimen considered the relatively short study duration while accounting for (i) the cumulative exposure to AHR ligands that humans experience over a lifetime, (ii) the significant difference in TCDD half-lives (1 to 11 years in humans compared to 8 to 12 days in mice [33,34] and (iii) the likelihood that individuals are exposed to additional AHR-activating compounds. This treatment regimen allowed hepatic TCDD levels to approach a steady state and effectively induced AHR-responsive genes, including those implicated in promoting reactive oxygen species (ROS) production. This study design has been used previously to show Pkm^ΔDRE^ mice exhibited greater susceptibility to TCDD-induced toxicity compared to WT controls [8].

### 4.6. LC-MS Analysis

Frozen liver samples were homogenized (Polytron PT2100, Kinematica, **Malters, Switzerland**) in 500 μL of HPLC-grade methanol, 200 μL of HPLC-grade water and 500 μL of HPLC-grade chloroform. Samples are vortexed for 10 min, then centrifuged at 4 °C 16,000× *g* for 15 min. The polar layer was then removed and dried under nitrogen gas. Extracted metabolites were then resuspended in 3% methanol containing 10 mM tributylamine (TBA).

Metabolite profiling was conducted by measuring relative abundance and high-resolution accurate mass spectrometry on a Xevo G2-XS QTof mass spectrometer (Waters, Eschborn, Germany) integrated with a Waters liquid chromatography system. Chromatographic separation utilized an Acquity HSS T3 column (1.8 μm particle size, 2.1 mm × 150 mm). A binary solvent system was applied for gradient elution. The mobile phase A consisted of LC/MS-grade water containing 3% LC/MS-grade methanol, 10 mM TBA, and 15 mM acetic acid (pH 5.0 ± 0.05). Mobile phase B was composed of LC/MS-grade methanol (100%). A constant flow rate of 300 μL/min was maintained and the linear gradient employed was as follows: 0–1.5 min 100% A, 1.5–4 min increase from 0 to 20% B, 4–6. min maintain 80% A and 20% B, 6–10.5 min increase from 20 to 55% B, 10–13 min increase from 55 to 95% B, 13–15 min maintain 5% A and 95% B, 15–15.1 min decrease from 95–0% B, 15.1–18 min equilibration at 100% A. The column temperature was maintained at 40 °C and sample volumes of 10 μL were injected. An 18 min full-scan method was used to acquire data with *m*/*z* scan range from 50 to 1000.

## Figures and Tables

**Table 1:** Number of differentially expressed genes (DEGs) at individual time points.

	2 h	4 h	8 h	12 h	24 h	48 h	72 h	96 h	120 h
Pkm^ΔDRE^ unique DEGs	651	1380	2709	176	259	162	96	371	224
WT unique DEGs	1743	511	240	214	183	266	368	442	138
Common DEGs	105	53	63	25	42	83	78	130	129

## Data Availability

Time-course hepatocytes RNA-seq data was deposited to GEO under the accession identifier GSE309637. The raw and processed data for LC-MS are available at Metabolomics Workbench under Accession ID: ST004256.

## Supplementary Materials

The supporting information can be downloaded at: https://www.mdpi.com/article/10.3390/ijms262210853/s1.
