# Supplementary material for: Loss of Dioxin Response Element-Mediated Induction of PKM2 Reprograms Hepatic Metabolism in Response to TCDD
Source: Int J Mol Sci. 2025 Nov 8;26(22):10853. doi: 10.3390/ijms262210853 (PMC12652037; doi:10.3390/ijms262210853)
Supplement: Supplementary file 1 [file ijms-26-10853-s001.zip › ijms-3939205-supplementary.pdf]

SUPPLEMENTARY INFORMATION

Loss of dioxin response element-mediated induction of PKM2 reprograms hepatic metabolism in response to TCDD

Karina Orlowska[1,2], Rance Nault[2,3] and Tim Zacharewski[1,2,*]

[1]Biochemistry & Molecular Biology, [2]Institute for Integrative Toxicology, Pharmacology & Toxicology, Michigan State University, East Lansing, MI 48824, USA

[*]Correspondence:
Tim Zacharewski
Michigan State University
Department of Biochemistry & Molecular Biology
Biochemistry Building
603 Wilson Road
East Lansing, MI 48824
tzachare@msu.edu

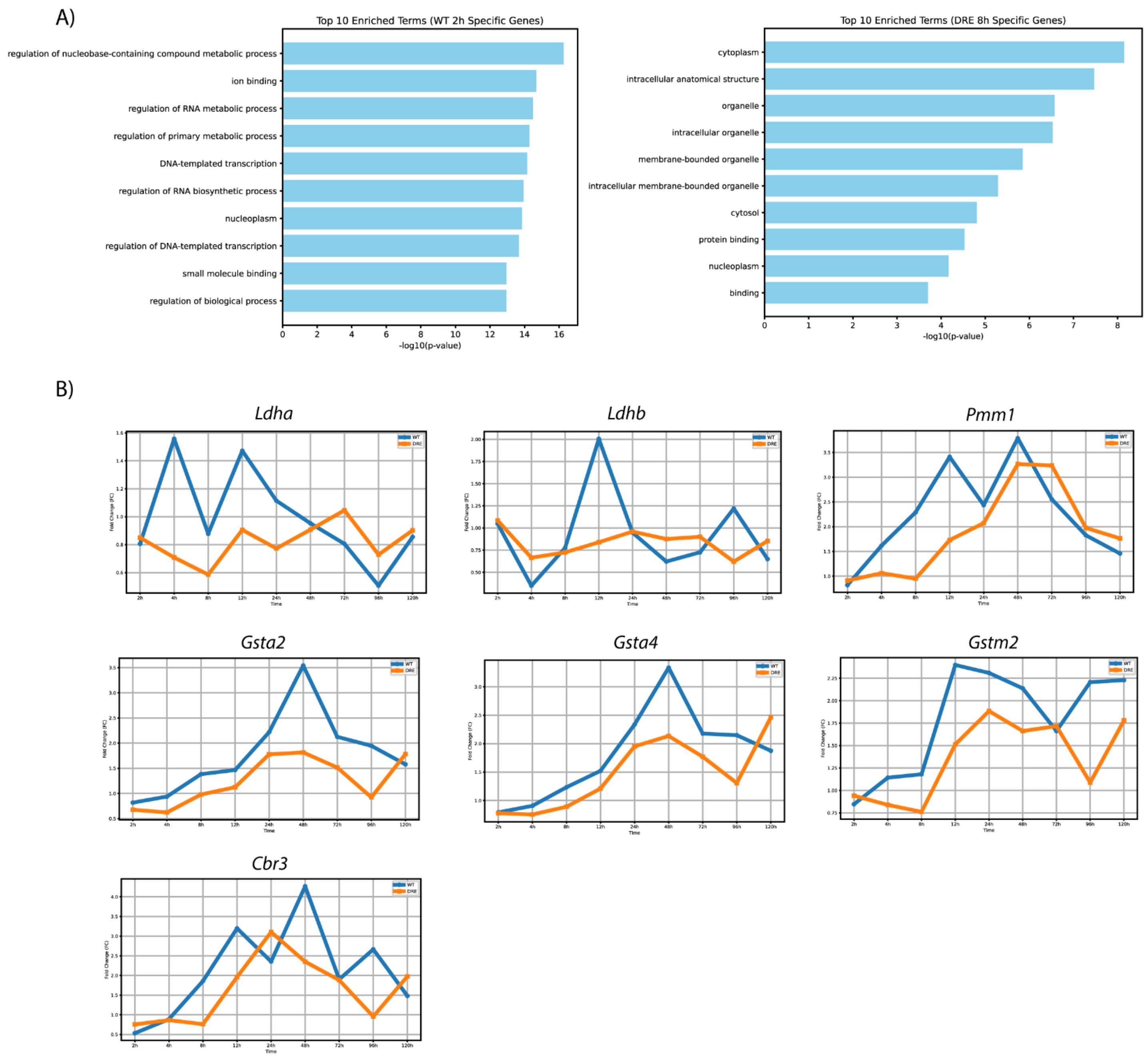

Supplementary Figure S1. Temporal changes in gene expression assessed by RNA-seq in primary mice hepatocytes treated with DMSO vehicle or 10 nM TCDD for 2, 4, 8, 12, 24, 48, 72, 96 and 120 hours. A) Functional enrichment of upregulated DEGs that were unique for 2h time point in WT and 8h time point in Pkm$^{\Delta DRE}$ mice. **B)** Examples of genes with disrupted responses to TCDD in PKM$^{\Delta DRE}$ mice relative to WT counterparts.

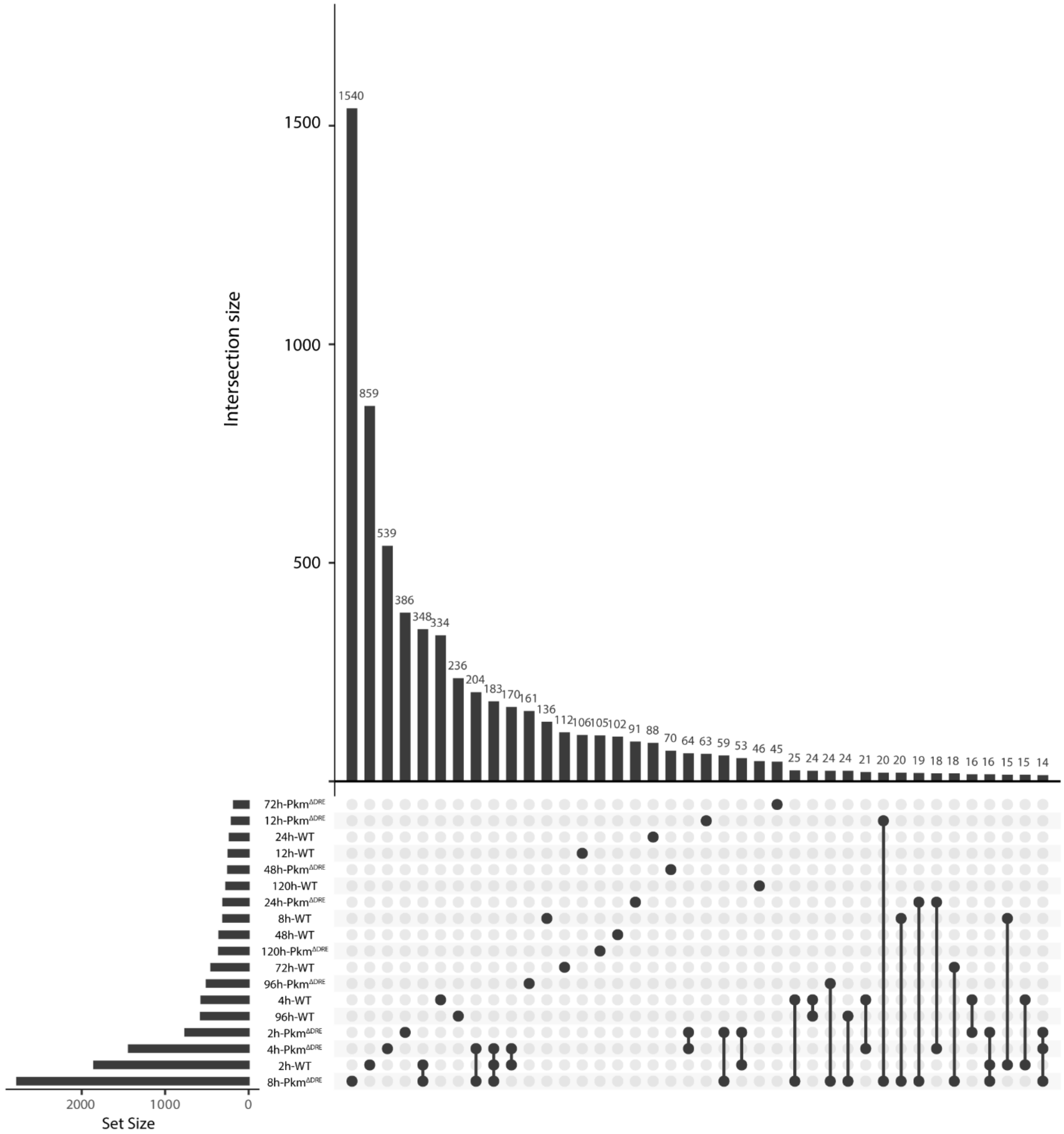

Supplementary Figure S2. Number of differentially expressed genes (DEGs) that are either unique to or shared between multiple time points in primary mouse hepatocytes exposed to 10 nM TCDD. Time-dependent gene expression was assessed in WT and Pkm$^{\Delta DRE}$ mouse hepatocytes treated with DMSO (vehicle) or 10 nM TCDD for 2, 4, 8, 12, 24, 48, 72, 96 and 120 hours**.**

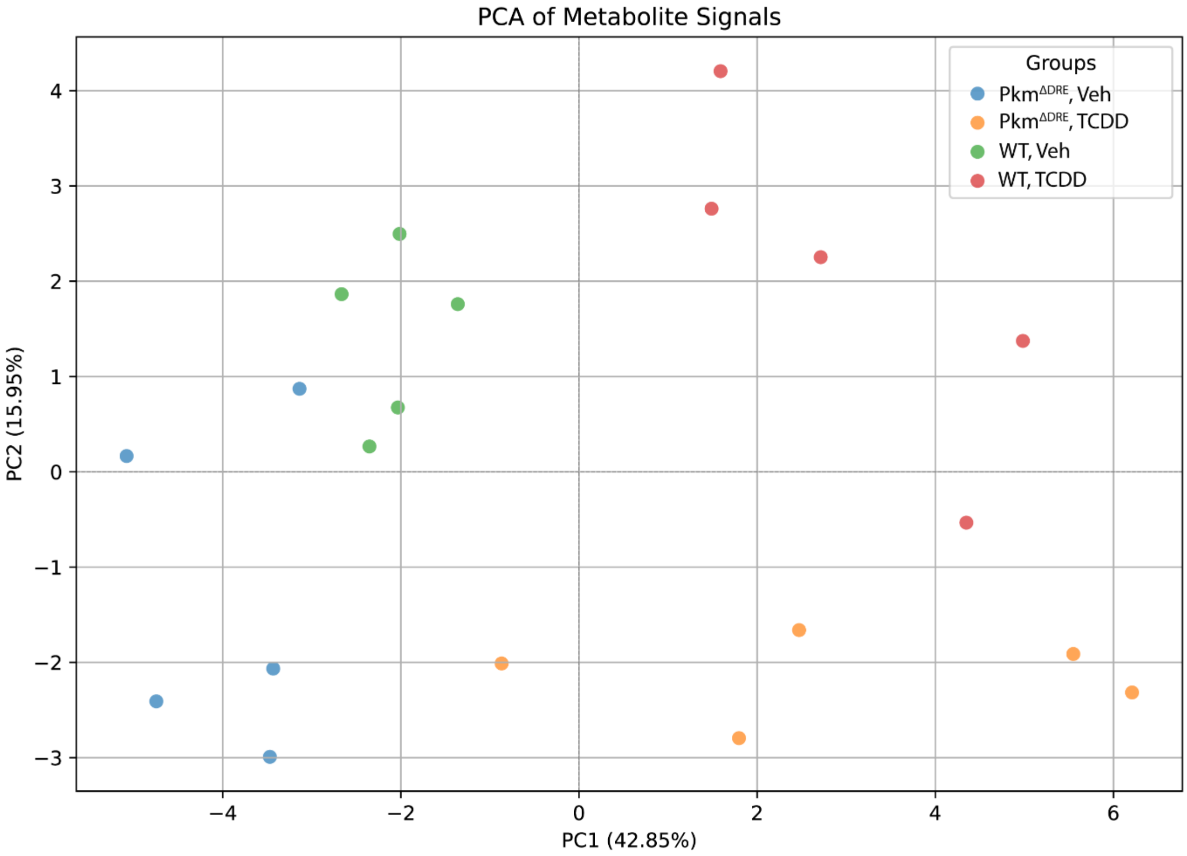

Supplementary Figure S3. Principal component analysis of 28 analyzed glycolysis, PPP, Ser/Gly/GSH pathway, TCA cycle, glutamine metabolites in liver extracts. WT and Pkm^{ΔDRE} mice were treated with sesame oil (vehicle) or 30 µg/kg TCDD every 4 day for 28 days. Livers were snap-frozen, and metabolites were extracted using 80% methanol. Samples were analyzed on a Xevo G2-XS Quadrupole Time of Flight mass spectrometer attached to a Waters Acquity UPLC (Waters) operated in negative-mode electrospray ionization

Supplementary Table S1. Top 10 metabolites contributing to principal components (PC) 1 and 2 based on absolute loading values.

Metabolite	PC1	Metabolite	PC2
GSSG	0.26227	Glycine	0.18551
Aspartate	0.25651	1,3-bisphospoglycerate	0.25937
Glycine	0.24734	Malate	0.34618
Serine	0.24728	Fumarate	0.34370
Glutamate	0.24240	Acetyl-CoA	0.31155
GAP/DAP	0.23968	Ketoglutarate	0.31057
Glutamine	0.23741	Pyruvate	0.24599
1,3-bisphospoglycerate	0.21932	2/3-phosphoglycerate	0.22549
Glucose-6-phosphate	0.20844	D-Sedoheptulose 7-phosphate	0.21782
Oxaloacetate	0.20635	GSH	0.19534

Supplementary Table S2: Primer sequences (5' -3') and product sizes for genes analyzed by qRT-PCR

Gene Symbol	Gene Name	Primer sequence		Product length	Specie
		Forward	Reverse		
Actb	Actin, beta	GCTACAGCTTCACCACCACA	TCTCCAGGGAGGAAGAGGAT	123	mouse
Gapdh	glyceraldehyde-3-phosphate dehydrogenase	GTGGACCTCATGGCCTACAT	TGTGAGGGAGATGCTCAGTG	125	mouse
Hprt	hypoxanthine guanine phosphoribosyl transferase	AAGCCTAAGATGAGCGCAAG	TTACTAGGCAGATGGCCACA	104	mouse
Pkm1	pyruvate kinase isoform 1	AGTCCTGGATGGAGCAGACT	TTCAAACAGCAGACGGTGGA	136	mouse
Pkm2	pyruvate kinase isoform 2	CATTACCAGCGACCCCACAG	CTCCTGCCAGACTTGGTGAG	102	mouse
